# Increased Risk of Sensorineural Hearing Loss as a Result of Exposure to Air Pollution

**DOI:** 10.3390/ijerph17061969

**Published:** 2020-03-17

**Authors:** Kuang-Hsi Chang, Stella Chin-Shaw Tsai, Chang-Yin Lee, Ruey-Hwang Chou, Hueng-Chuen Fan, Frank Cheau-Feng Lin, Cheng-Li Lin, Yi-Chao Hsu

**Affiliations:** 1 Department of Medical Research, Tungs’ Taichung Metroharbor Hospital, Taichung 43503, Taiwan; kuanghsichang@gmail.com (K.-H.C.); t11578@ms.sltung.com.tw (H.-C.F.); 2 Graduate Institute of Biomedical Sciences, China Medical University, Taichung 40402, Taiwan; rhchou@mail.cmu.edu.tw; 3 General Education Center, Jen-Teh Junior College of Medicine, Nursing and Management, Miaoli 35664, Taiwan; 4 Department of Otolaryngology, Tungs’ Taichung Metroharbor Hospital, Taichung 43503, Taiwan; tsaistella111@gmail.com; 5 College of Medicine, The School of Chinese Medicine for Post Baccalaureate, I-Shou University (Yancho Campus), Kaohsiung 84001, Taiwan; mikeleefafa@gmail.com; 6 Department of Chinese Medicine, E-DA Hospital, Kaohsiung 82445, Taiwan; 7 Department of Chinese Medicine, E-DA Cancer Hospital, Kaohsiung 82445, Taiwan; 8 Center for Molecular Medicine, China Medical University Hospital, Taichung 40402, Taiwan; 9 Department of Biotechnology, Asia University, Taichung 41354, Taiwan; 10 Department of Pediatrics, Department of Medical Research, Tungs’ Taichung Metroharbor Hospital, Taichung 43503, Taiwan; 11 Department of Rehabilitation, Jen-Teh Junior College of Medicine, Nursing and Management, Miaoli 35664, Taiwan; 12 Department of Thoracic Surgery, Chung Shan Medical University Hospital, Taichung 40201, Taiwan; frnklin@gmail.com; 13 Management Office for Health Data, China Medical University Hospital, Taichung 40402, Taiwan; orangechengli@gmail.com; 14 Institute of Biomedical Sciences, Mackay Medical College, New Taipei City 252, Taiwan

**Keywords:** air pollution, sensorineural hearing loss, National Health Insurance Research Database (NHIRD), hazard ratio

## Abstract

Whether exposure to air pollution is associated with developing sensorineural hearing loss (SHL) remains controversial. Using data from the National Health Insurance Research Database, we recruited a total of 75,767 subjects aged older than 20 years with no history of SHL from 1998 to 2010, and they were followed up until SHL was observed, they withdrew from the National Health Insurance program, or the study ended. The subjects were evenly exposed to low-level, mid-level, and high-level carbon monoxide (CO) and nitrogen dioxide (NO_2_). The incidence rate ratio of SHL for patients exposed to high-level CO was 1.24 (95% confidence interval (CI) = 1.14–1.36). The NO_2_ pollutants increased the incidence rate ratios of SHL in mid-level NO_2_ and high-level NO_2_ exposures by 1.10 (95% CI = 1.10–1.32) and 1.36 (95% CI = 1.24–1.49) times, respectively. The adjusted hazard ratio (adj. HR) of SHL in patients exposed to high-level CO was 1.45 (95% CI = 1.31–1.59), relative to that of patients exposed to low-level CO. Compared to patients exposed to low-level NO_2_, patients exposed to mid-level NO_2_ (adj. HR = 1.40, 95% CI = 1.27–1.54) and high-level NO_2_ (adj. HR = 1.63, 95% CI = 1.48–1.81) had a higher risk of developing SHL. The increased risk of SHL following the increased concentrations of air pollutants (CO and NO_2_) was statistically significant in this study. In conclusion, the subjects’ exposure to air pollution exhibited a significantly higher risk of developing SHL in Taiwan.

## 1. Introduction

In developing and developed countries, air pollution has already become an important health issue. Several factors, such as urbanization, industrialization, heavy traffic, or establishment of thermal power stations, result in air pollution in several countries. Primarily, lung cancer is considered the major severe disease caused by air pollution [1,2]. Notably, accumulating evidence shows that chronic exposure to air pollution is associated with not only respiratory diseases but also non-lung cancers [3,4] and cardiovascular [5,6], inflammatory [7,8], and neurodegenerative diseases [9,10] including age-related macular degeneration (AMD) [11].

Sensorineural hearing loss (SHL) is defined as the damage, degeneration, or loss of the cochlear hair cells (HCs) [12,13] or spiral ganglion neurons [14,15,16] in the auditory system. The majority of SHL patients are diagnosed with congenital SHL including syndromic and non-syndromic SHL. It has been shown that a total of 70–100 genetic loci are associated with SHL. These genetic loci are usually found in genes responsible for signal transduction, membrane ion channel expression, HC development, and aging processes [13]. Most of the genes are directly related to the function of the outer HCs in fine-tuning incoming sound waves. Currently, hearing aids and cochlear implants are clinically beneficial in restoring the hearing function of patients with congenital SHL. However, Food and Drug Administration-approved drugs used to treat congenital or degenerative SHL are not yet available.

Several etiological factors can cause SHL, such as loud noise [17,18], viral infection [14,19], genetic mutations [20,21], accidental events [22,23], ototoxicity [24,25,26], autoimmune diseases [27] and unknown illness-induced sudden SHL [28,29]. Several diseases such as hypertension (HT) [30,31], diabetes mellitus (DM) [32,33], stroke [34], chronic kidney disease (CKD) [35,36], ischemic heart disease (IHD) [37,38], alcoholism [39,40], nicotine dependence [41,42], asthma [43,44,45], chronic obstructive pulmonary disease (COPD) [46], and rheumatoid arthritis (RA) [47] are associated with SHL. Notably, some of these diseases can cause defects in the vascular system and microcirculation in the cochlea, subsequently resulting in hypoxia [48,49,50]. Hypoxia has been demonstrated to damage the HCs and neurons in the inner ear [48,50]. Furthermore, it has been shown that hypoxia-inducible factor-1α is upregulated by cobalt chloride-induced hypoxia in noise-induced hearing loss [51].

Accumulating evidence using the analyses of the National Health Insurance Research Database (NHIRD) in Taiwan shows that chronic exposure to air pollution increases the risk of neurodegenerative diseases including Parkinson’s disease (PD) [9], dementia [10], and AMD [11]. Although air pollution has been considered a risk factor for several diseases, its association with hearing disorders has been less studied. A previous study conducted in Busan, Korea, showed that air pollution is possibly associated with sudden SHL [52]. However, whether air pollution is associated with SHL remains controversial. Therefore, it is significantly interesting to hypothesize that air pollution may also be associated with SHL development. In this study, we focused on investigating the two major traffic-related air pollutants: nitrogen dioxide (NO_2_) and carbon monoxide (CO). We analyzed the data from a longitudinal cohort study using the Taiwan National Health Insurance (NHI) data to assess the significant effects of these traffic-related air pollutants to SHL.

## 2. Subjects and Methods

### 2.1. Data Source and Study Subjects

A single-payer Taiwan NHI program was launched in March 1995. Approximately 99% of Taiwan’s population are included in this program. The NHIRD consists of insured’s inpatients and outpatient claims, medications, and treatment data. In the present study, we utilized the Longitudinal Health Insurance Database which is the health database of a million beneficiaries randomly selected from the NHIRD. The identification information was encrypted to protect the patients’ personal privacy. The disease codes were identified according to the International Classification of Disease, Ninth Revision, Clinical Modification (ICD-9-CM). We recruited subjects aged older than 20 years with no history of SHL from 1998 to 2010, and they were followed up until SHL was observed, they withdrew from the NHI program, and the study ended (December 2011).

### 2.2. Exposure Measurement

The data regarding the air pollutants were collected from 74 ambient air quality monitoring stations across Taiwan. The air quality data are managed by Taiwan Environmental Protection Administration. The annual concentrations of NO_2_ and CO from 1998 to 2010 were grouped into the following three levels according to tertiles: the low-, mid-, and high-level groups. The NO_2_ concentrations of the low-, mid-, and high-level groups were <19.53 (ppb), 19.53–25.66 (ppb), and >25.66 (ppb), respectively. Moreover, the CO concentrations of the low-, mid-, and high-level groups were <0.61 (ppm), 0.61–0.76 (ppm), and >0.76 (ppm), respectively. To analyze the exposures across a 13 year period (from 1998 to 2010), we calculated the annual average of pollutants from baseline to the date of hearing loss occurrence.

### 2.3. Main Outcome and Covariates

Hearing loss (ICD-9-CM code 389.10–389.12 and 388.01) was the primary event in this study. Besides age and sex, we also considered the insurance fee and area of residence of the subjects. The common comorbidities including HT (ICD-9-CM codes 401–405) [30,31], DM (ICD-9-CM code 250) [32,33], stroke (ICD-9-CM codes 430–438) [34], head injury (ICD-9-CM codes 850–854) [22,23], CKD (ICD-9-CM code 585) [35,36], IHD (ICD-9-CM codes 410–414) [37,38], alcoholism (ICD-9-CM codes 305.0 and 303) [39,40], nicotine dependence (ICD-9-CM code 305.1) [41,42], asthma (ICD-9-CM code 493) [43,44,45], COPD (ICD-9-CM codes 490–492, 494, and 496) [46], and RA (ICD-9-CM code 714) [47] were considered as potential confounders.

### 2.4. Statistical Analysis

We show the demographic distribution as a percentage. The incidence density rates of hearing loss were calculated, and the incidence rate ratio (IRR) was assessed by Poisson regression. We estimated the hazard ratios using the multivariate Cox proportional hazard model, adjusting for age, sex, insurance fee, urbanization, and comorbidities.

## 3. Results

We recruited 75,767 subjects in the present study. Table 1 shows the distribution of the demographic characteristics of the study sample. The subjects’ mean age was 54.1 (±11.1) years with a mean age of 11.1 (±2.2) years the following years. Moreover, 45.3% of the subjects were men. The subjects were evenly exposed to low-, mid-, and high-level CO and NO_2_. Most of the subjects lived in moderately urbanized areas (33.1%) and highly urbanized areas (32.5%). Table 2 shows that the top three comorbidities with high incidence rates were HT (57.5%), DM (20.6%), stroke (9.1%), head injury (9.8%), CKD (5.3%), IHD (34.6%), alcoholism (1.4%), nicotine dependence (2.2%), asthma (17.5%), COPD (34.5%), and RA (0.5%).

The IRRs of SHL in different air pollutant exposure levels are presented in Table 3. Subjects exposed to low-level air pollutants were assigned to the reference group. The IRR of SHL in subjects exposed to high-level CO was 1.24 (95% confidence interval (CI) = 1.14–1.36). The NO_2_ pollutants increased the incidence of SHL in subjects exposed to mid-level NO_2_ and high-level NO_2_ by 1.10 (95% CI = 1.10–1.32) and 1.36 (95% CI = 1.24–1.49) times, respectively. From our analyses, the CO levels in highly urbanized region, moderately urbanized region, boomtown, and others were 0.82 ± 0.31, 0.69 ± 0.24, 0.71 ± 0.22, and 0.58 ± 0.21, respectively (Table 3). Furthermore, the NO_2_ levels in highly urbanized region, moderately urbanized region, boomtown, and others were 24.69 ± 6.65, 21.83 ± 6.45, 23.54 ± 5.53, 17.87 ± 6.37, respectively (Table 3). Our results suggest that the distribution of CO and NO_2_ levels are highly correlated with the urbanization.

According to Table 4, the adjusted hazard ratio (adj. HR) of SHL in subjects exposed to high-level CO was 1.45 (95% CI = 1.31–1.59), relative to that of subjects exposed to low-level CO. Compared to subjects exposed to low-level NO_2_, subjects exposed to mid-level NO_2_ (adj. HR = 1.40, 95% CI = 1.27–1.54) and high-level NO_2_ (adj. HR = 1.63, 95% CI = 1.48–1.81) had a higher risk of developing SHL. Moreover, the increased risk of SHL following the increased concentrations of air pollutants (CO and NO_2_) was statistically significant in this study. In the present study, we found that the incidence rate ratio of SHL in the highest level of CO (IRR = 1.24, 95%CI = 1.14–1.36) was significantly higher than that in low level of CO (Table 4). Furthermore, we found that the incidence rate ratio of SHL in the mid-level (IRR = 1.21, 95% CI = 1.10–1.32) and highest level of NO_2_ (IRR = 1.36, 95% CI = 1.24–1.49) was significantly higher than that in low level of NO_2_ (Table 4).

**Figure 1:**
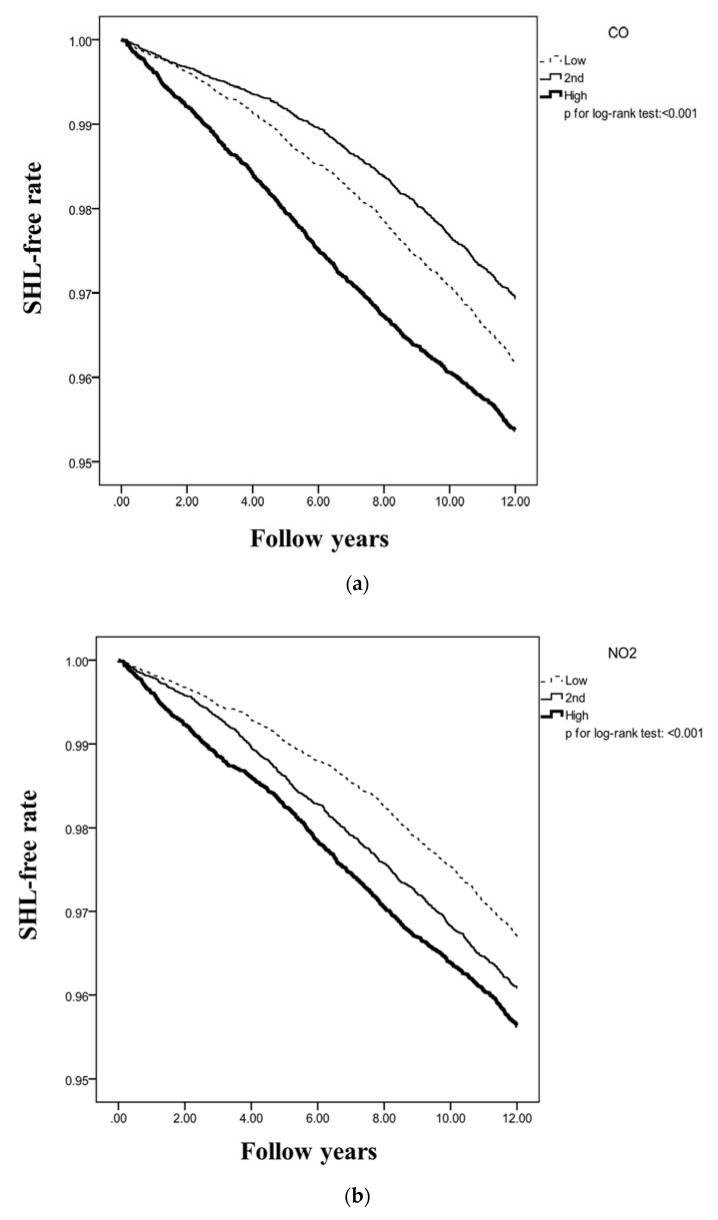
Probability free of SHL among three levels (Low, 2nd or High level) of pollutants concentrations: (**a**) CO; (**b**) NO_2_.

According to Table 5, the aHR of hearing loss in the high CO concentration group relative to the low CO concentration group was 1.45 (95% CI, 1.31–1.59). Patients in the mid-NO_2_ level (aHR = 1.40, 95% CI = 1.27–1.54) and high NO_2_ level (aHR = 1.63, 95% CI = 1.48–1.81) groups had a higher risk of hearing loss compared to patients in the low NO_2_ level group. The increased concentrations of the air pollutants (i.e., CO and NO2) enhancing the risk of hearing loss was statistically significant. Moreover, continuous analyses based on the subjects’ sex and age (subjects aged 50 years) to assess the association between air pollution and SHL were performed. Our data suggest that both male (aHR = 1.25, 95% CI = 1.03–1.52) and female (aHR = 1.49, 95% CI = 1.20–1.85) subjects have a significantly high incidence of SHL when they are exposed to CO pollution. Moreover, female subjects had a significantly high incidence of SHL when exposed to NO2 (aHR = 1.01, 95% CI = 1.001–1.02). Furthermore, elderly subjects aged greater than 50 years had a significantly higher risk of SHL (CO: aHR = 1.37, 95% CI = 1.17–1.62) when they were exposed to CO pollution. Additionally, we provided the probability-free prediction of SHL among the three levels of CO and NO2 concentrations (Figure 1).

## 4. Discussion

Although air pollution has been considered a risk factor for several diseases, its association with hearing disorders has been less studied. In this study, we hypothesized that air pollution may also be associated with SHL development. We focused on investigating the two major traffic-related air pollutants: nitrogen dioxide (NO2) and carbon monoxide (CO). We analyzed the data from a longitudinal cohort study using Taiwan National Health Insurance (NHI) data to assess the significant effects of these traffic-related air pollutants to SHL. Our results revealed that the subjects who were chronically exposed to air pollution were at a significantly increased risk of developing SHL. Based on the longitudinal human data, our results suggest that NO_2_ and CO could have potential adverse effects on SHL. Furthermore, we showed that long-term exposure to the highest quartile of NO_2_ significantly increased the risk of SHL by almost 1.63 fold, even after adjusting the confounding factors. Additionally, exposure to the highest quartile of CO also increased the risk of SHL by 1.45 fold. Nitrogen dioxide and CO result in cellular damages through different mechanisms. Nitrogen dioxide is a reactive nitrogen species (RNS). Reactive nitrogen species and reactive oxygen species cause cell apoptosis. On the contrary, CO is considered toxic, as it prevents the blood system from effectively carrying oxygen around the body, specifically to the vital organs such as the heart and brain. However, any report showing the association between CO and SHL does not exist yet. The present study suggests that exposure to CO and NO_2_ has several adverse effects on the auditory system.

Several studies investigating the clinical effects of air pollution on different organ systems in the human body have been conducted [4,7,8,9,11,53]. Using similar datasets, we have previously shown that CO and NO_2_ were associated with AMD [11], dementia [53], and PD [9]. Compared with the lowest exposure group, the adj. HRs for AMD and dementia were 1.91 and 1.5 and 1.84 and 1.61 for the highest quartile of NO_2_ exposure and the highest quartile of CO exposure, respectively. Based on the adj. HRs, our data indicated that these two pollutants have similar adverse effects on SHL, AMD, and dementia. On the contrary, either NO_2_ or CO is not associated with the incidence of PD. However, particulate matter 10 (PM_10_) exposure can have significant adverse effects on PD development. Similar to AMD and dementia, SHL is also an age-related disease that primarily affects the cochlear hair cells and spiral ganglion neurons.

The onset of SHL has been associated with several risk factors including inflammation and oxidative stress. Our data further suggest that RNS might also contribute to SHL progression. Nitrogen dioxide is one of the most important components of traffic-related air pollution. It can be hydrolyzed to nitrous and nitric acid, both of which can induce inflammation by oxidative stress and lipid peroxidation. It has been suggested that gentamicin application intratympanally can result in a hearing threshold shift beginning on the second day after gentamicin application. This hearing impairment is simultaneously associated with an increased NO_2_^−^ (a stable oxidation product of NO_2_) concentration in the lateral wall, indicating that NO_2_^−^ contributes to gentamicin-induced hearing impairment [54].

Although the primary route of exposure to NO_2_ is via inhalation, studies assessed not only the association between the respiratory system and NO_2_ exposure but also the association between the cardiovascular [55,56] and neurological systems and NO_2_ exposure [57]. Previously, air pollution has been reported to be a risk factor for sudden SHL [52]. The major differences between our results and the reports of Lee et al. [52] are as follows: (1) our study was the first study to use a large longitudinal cohort from 1998 to 2010 to demonstrate the significant association between SHL and high levels of ambient NO_2_ and CO. (2) According to the report of Lee et al. [52], they utilized the data from the daily patient numbers admitted to the hospital with sudden SHL in 2015 and demonstrated that in Busan, Korea, there is a statistically significant weak association between the daily numbers of patients admitted to the hospital with sudden SHL and the exposure of mean daily PM_10_ and PM_2.5_ concentrations [52].

It has been suggested that the risk of SHL is also significantly associated with factory workers who are co-exposed to noise and heavy metals or organic solvents [58]. Co-exposure to noise and heavy metal or toxic pollution results in a worse SHL compared with isolated exposures [59]. In the present study, both CO and NO_2_ levels are relatively higher in highly urbanized region and boomtown than in other areas (Table 3). Notably, both noise and air pollution may damage the cochlear HCs via oxidative stress [60], hypoxia-induced reactive oxygen species, and HC damage [48]. Although we found a statistically significant association between air pollution and subsequent SHL, the present study had the following limitations that need to be considered: (1) The noise levels in the studied area and the genetic variants in the studied subjects, which are considered as important risk factors for SHL, were not clarified in the present study, and without the noise-level data, the scope and significance of the study are limited. (2) The study conducted in the assigned residential area was according to the subject’s condition which was associated with the treatment of acute upper respiratory infections in a clinic or hospital, resulting in the underestimation of every subject’s exposure level to each air pollutant in the studied area. However, our findings are still significant and require further investigation. (3) In the present study, we used the ICD-9-CM codes 389.10–389.12 and 388.01 for data acquisition of age-related hearing loss and SHL. However, we did not include the ICD-9-CM codes of sudden hearing loss (388.02) and conductive hearing loss (389.00 and 389.06) considering that only 3.6% (2724 SHL subjects/75,767 subjects) of the subjects were identified as SHL patients in this study (Table 1).

## 5. Conclusions

In conclusion, we linked the national health database and air quality database to report NO_2_ and CO as risk factors for SHL. Our results indicate that the highest quartile of each pollutant could increase the risk of SHL by almost 1.63 and 1.45 fold, respectively. Notably, a mid-level exposure to NO_2_, but not CO, could also significantly contribute to SHL development by 1.40 fold.

## Figures and Tables

**Table 1:** Distribution of the demographic data of the study participants.

Covariates	Categories	Hearing Loss (*n* = 2724)		Without Hearing Loss (*n* = 73,043)		*p*	Total (*n* = 75,767)	
Age	Mean (SD)	60.8	(11.6)	53.9	(11.0)	<0.001	54.1	(11.1)
Follow years	Mean (SD)	6.3	(3.4)	11.3	(1.9)	<0.001	11.1	(2.2)
Male		1569	57.6	32,791	44.9	<0.001	34,360	45.3
Insurance fee (NTD)	>17,400	1148	42.1	22,948	31.4	<0.001	24,096	31.8
	17,400–19,200	884	32.5	25,686	35.2		26,570	35.1
	>19,200	692	25.4	24,409	33.4		25,101	33.1
Urbanization	Highly	815	29.9	23,838	32.6	<0.001	24,653	32.5
	Moderately	921	33.8	24,186	33.1		25,107	33.1
	Boomtown	362	13.3	11,817	16.2		12,179	16.1
	Others	626	23.0	13,202	18.1		13,828	18.3
CO	Low	918	33.7	24,800	34.0	<0.001	25,718	33.9
	2nd	717	26.3	24,187	33.1		24,904	32.9
	High	1089	40.0	24,056	32.9		25,145	33.2
	Mean (SD)	0.72	(0.27)	0.71	(0.27)	0.313	0.71	(0.27)
NO_2_	Low	834	30.6	26,178	35.8	<0.001	27,012	35.7
	2nd	909	33.4	23,839	32.6		24,748	32.7
	High	981	36.0	23026	31.5		24,007	31.7
	Mean (SD)	22.10	(7.32)	22.31	(6,76)	0.129	22.31	(6.78)

CO: carbon monoxide; NO_2_: nitrogen dioxide; NTD: new Taiwan dollar.

**Table 2:** Distribution of the comorbidities of the study participants.

*n*, %	Hearing Loss (*n* = 2724)		Without Hearing Loss (*n* = 73,043)		*p*	Total (*n* = 75,767)	
HT	1728	63.4	41,813	57.2	<0.001	43,541	57.5
DM	519	19.1	15,105	20.7	0.042	15,624	20.6
Stroke	208	7.6	6683	9.1	0.008	6891	9.1
Head injury	267	9.8	7135	9.8	0.980	7402	9.8
CKD	139	5.1	3846	5.3	0.742	3985	5.3
IHD	1201	44.1	25,007	34.2	<0.001	26,208	34.6
Alcoholism	26	1.0	1019	1.4	0.064	1045	1.4
Nicotine	32	1.2	1599	2.2	<0.001	1631	2.2
Asthma	495	18.2	12,779	17.5	0.375	13,274	17.5
COPD	1208	44.3	24,954	34.2	<0.001	26,162	34.5
RA	11	0.4	352	0.5	0.661	363	0.5

HT: hypertension; DM: diabetes mellitus; CKD: chronic kidney disease; IHD: ischemic heart disease; Nicotine: nicotine dependence; COPD: chronic obstructive pulmonary disease; RA: rheumatoid arthritis.

**Table 3:** Distributions of NO_2_ and CO among urbanization zones.

*n*, %		Highly Urbanized (*n* = 24,653)		Moderately Urbanized (*n* = 25,107)		Boomtown (*n* = 12,179)		Others (*n* = 13,828)		Total (*n* = 75,767)	
CO	Low	4891	19.8	8729	34.8	3828	31.4	8270	59.8	25,718	33.9
	2nd	6718	27.3	9220	36.7	5056	41.5	3910	28.3	24,904	32.9
	High	13,044	52.9	7158	28.5	3295	27.1	1648	11.9	25,145	33.2
	Mean (SD)	0.82 (0.31)		0.69 (0.24)		0.71 (0.22)		0.58 (0.21)		0.71 (0.27)	
NO_2_	Low	5709	23.2	9841	39.2	2912	23.9	8550	61.8	27,012	35.7
	2nd	5851	23.7	9334	37.2	5627	46.2	3936	28.5	24,748	32.7
	High	13,093	53.1	5932	23.6	3640	29.9	1342	9.7	24,007	31.7
	Mean (SD)	24.69 (6.65)		21.83 (6.45)		23.54 (5.33)		17.87 (6.37)		22.31 (6.78)	

**Table 4:** Incidence and incidence rate ratio of hearing loss for the three levels of air pollutant exposure.

Pollutants	Levels	*n* of HL	Follow Years	IR	IRR	95%CI
CO	Low	918	287,414	3.19	1.00	
	2nd	717	282,256	2.54	0.79	0.72–0.88
	High	1089	274,908	3.96	1.24	1.14–1.36
NO_2_	Low	834	304,577	2.74	1.00	
	2nd	909	275,599	3.30	1.21	1.10–1.32
	High	981	264,401	3.71	1.36	1.24–1.49

PY: person-years; *n* of HL: number of patients with hearing loss; IR: incidence rate (per 1000 person-years); IRR: incidence rate ratio.

**Table 5:** Adjusted HR of hearing loss in the moderate and high concentration groups compared to that in the low concentration group.

Pollutants	Levels	All			Sex-Specific Risk			Age-Specific Risk		
		Adjusted HR	95%CI	*p*	Adjusted HR	95%CI	*p*	Adjusted HR	95%CI	*p*
CO	continuous	1.35	1.17–1.56	<0.001	1.25^male^	1.03–1.52	<0.001	1.30^<=50^	0.97–1.74	0.084
	2nd versus Low	0.90	0.82–1.00	0.050	0.88^male^	0.77–1.01	0.064	1.00^<=50^	0.81–1.23	0.995
	High versus Low	1.45	1.31–1.59	<0.001	1.38^male^	1.22–1.57	<0.001	1.65^<=50^	1.35–2.03	<0.001
	continuous				1.49^female^	1.20–1.85	<0.001	1.37^>50^	1.17–1.62	<0.001
	2nd versus Low				0.94^female^	0.81–1.10	0.459	0.88^>50^	0.79–0.99	0.034
	High versus Low				1.53^female^	1.32–1.77	<0.001	1.394^>50^	1.25–1.55	<0.001
NO_2_	continuous	1.01	1.001–1.01	0.024	1.01^male^	1.00–1.01	0.215	1.01^<=50^	1.00–1.02	0.168
	2nd versus Low	1.40	1.27–1.54	<0.001	1.38^male^	1.21–1.56	<0.001	1.45^<=50^	1.18–1.79	<0.001
	High versus Low	1.63	1.48–1.81	<0.001	1.56^male^	1.36–1.78	<0.001	1.92^<=50^	1.55–2.37	<0.001
	continuous				1.01^female^	1.001–1.02	0.031	1.01^>50^	1.00–1.01	0.057
	2nd versus Low				1.41^female^	1.22–1.64	<0.001	1.39^>50^	1.25–1.55	<0.001
	High versus Low				1.73^female^	1.49–2.02	<0.001	1.56^>50^	1.40–1.75	<0.001

Adjusted HR: adjusted hazard ratio in the multivariate analysis after adjusting for age, sex, insurance fee, urbanization, HT, DM, stroke, head injury, CKD, IHD, alcoholism, nicotine dependence, asthma, and COPD.

## Data Availability

The authors confirm that all data underlying the findings are fully available without restriction. The dataset is owned by the Taiwan National Health Research Institutes (NHRI). Requests for the dataset may be sent via an e-mail to the NHRI at nhird@nhri.org.tw or call at +886-037-246166 ext. 33603 for immediate service. Office Hours: Monday–Friday, 8:00–17:30 (UTC+8).

## Abbreviations

AMD	Age-related macular degeneration
CO	Carbon monoxide
LHID	Longitudinal Health Insurance Database
NHIRD	National Health Insurance Research Database
NHI	National Health Insurance
NO2	Nitrogen dioxide
PD	Parkinson’s disease
PM10	Particulate matter 10
RNS	Reactive nitrogen species
SHL	Sensorineural hearing loss
